# Compliance, Palatability and Feasibility of PALEOLITHIC and Australian Guide to Healthy Eating Diets in Healthy Women: A 4-Week Dietary Intervention

**DOI:** 10.3390/nu8080481

**Published:** 2016-08-06

**Authors:** Angela Genoni, Johnny Lo, Philippa Lyons-Wall, Amanda Devine

**Affiliations:** 1 School of Medical and Health Sciences, Edith Cowan University, Perth 6027, WA, Australia; P.Lyons-Wall@ecu.edu.au (P.L.-W.); a.devine@ecu.edu.au (A.D.); 2 School of Science, Edith Cowan University, Perth 6027, WA, Australia; J.Lo@ecu.edu.au

**Keywords:** Paleolithic, AGHE, cardiovascular, obesity, metabolic

## Abstract

(1) Background/Objectives: The Paleolithic diet has been receiving media coverage in Australia and claims to improve overall health. The diet removes grains and dairy, whilst encouraging consumption of fruits, vegetables, meat, eggs and nuts. Our aim was to compare the diet to the Australian Guide to Healthy Eating (AGHE) in terms of compliance, palatability and feasibility; (2) Subjects/Methods: 39 healthy women (age 47 ± 13 years, BMI 27 ± 4 kg/m^2^) were randomised to an ad-libitum Paleolithic (*n* = 22) or AGHE diet (*n* = 17) for 4-weeks. A food checklist was completed daily, with mean discretionary consumption (serves/day) calculated to assess compliance. A 12-item questionnaire was administered post intervention to assess palatability and feasibility; (3) Results: The AGHE group reported greater daily consumption of discretionary items (1.0 + 0.6 vs. 0.57 + 0.6 serves/day, *p* = 0.03). Compared to the AGHE group, the Paleolithic group reported a significantly greater number of events of diarrhoea (23%, 0%, *p* = 0.046), costs associated with grocery shopping (69%, 6% *p* < 0.01) and belief that the diet was not healthy (43%, 0% *p* < 0.01); (4) Conclusions: Compliance to both diets was high but the potential side effects and increased cost suggest that the Paleolithic diet may not be practical in clinical/public health settings. Further studies are required to assess longer term feasibility.

## 1. Introduction

The Paleolithic diet has been receiving a high level of media coverage in Australia [1,2]. The diet recommends the elimination of processed foods and sugars, however, it is contrary to the advice provided by the Australian Guide to Healthy Eating (AGHE) [3], given that it excludes two major food groups, grains and dairy. A limited number of smaller studies have reported benefits to cholesterol, weight control and glucose metabolism over short-term periods [4,5,6,7]. Despite potential health benefits, a Swedish study found that women following a Paleolithic diet reported difficulty changing food habits and longed for restricted food after a period of time [8]. Similarly, Manheimer et al. [9] identified the potential barrier of restricting the consumption of two entire food groups. Metzgar et al. [10] reported that in the US, a 9.3% increase in income would be required to follow a Paleolithic diet and provide sufficient nutrition to meet all the recommended dietary intakes (except for calcium). To the best of our knowledge, there is no literature on the palatability and feasibility of the Paleolithic diet in an Australian setting. Therefore, our aim was to compare the Paleolithic diet to the AGHE in terms of compliance, palatability and feasibility.

## 2. Materials and Methods

Thirty-nine healthy women (age 47 ± 13 years, BMI 27 ± 4 kg/m^2^) were randomised to an ad-libitum Paleolithic (*n* = 22) or AGHE diet (*n* = 17) as part of a larger study examining the cardiovascular and metabolic impacts of the diets, for a 4-week period [11]. Sample size calculations were performed α-priori and based on expected reductions in total plasma cholesterol, as reported in our previous publication [11]. The study was approved by the Edith Cowan University Human Research Ethics Committee (Project 10176) and registered on the Australia and New Zealand Register of Clinical Trials (ACTRN12615000246583). All participants provided informed consent prior to commencement of the intervention. In brief, pre and post-intervention biochemical and anthropometric measures, in addition to three day weighed food records, were collected as part of the study. To assess compliance to the diets, subjects completed a food checklist on a daily basis, where allowable foods on the dietary plan were ticked when eaten. Additional food items that were consumed but not on the checklist were recorded separately and subjects were asked to provide a full description of the food item and the serve size. All checklists were returned and checked for completeness. Energy value of the food consumed was calculated from the serve size and divided by 600 kJ to determine the number of extra food serves, consistent with the determination of a discretionary food item under the AGHE [3]. For the Paleolithic group an extra food was defined as any food which included cereals and grains, legumes, dairy and added refined sugars.

A 12-item questionnaire examined palatability and feasibility of the dietary intervention at completion of the study. A question relating to overall taste of the diet was used to assess palatability; feasibility was assessed with 8 questions relating to adaptation to the dietary pattern, ability to maintain the diet in social settings, feelings of wellbeing, cost of ingredients and whether the subject would recommend the dietary pattern in the future. Overall satiety of the diet was assessed using a 5-point Likert scale that related to the overall level of satisfaction or fullness with a range from feeling very full to still feeling very hungry. One participant in each group did not return the questionnaire for assessment. Daily adverse events and associated symptoms were recorded on the checklist.

To determine the difference in extras/discretionary serves consumed between groups, data was examined for normality and an independent *t*-test was used. The Fisher’s exact test was used to assess differences in adverse events and responses to the palatability and feasibility questionnaire between groups. A probability of *p* < 0.05 was taken as statistically significant.

## 3. Results

### 3.1. Compliance

**Figure 1:**
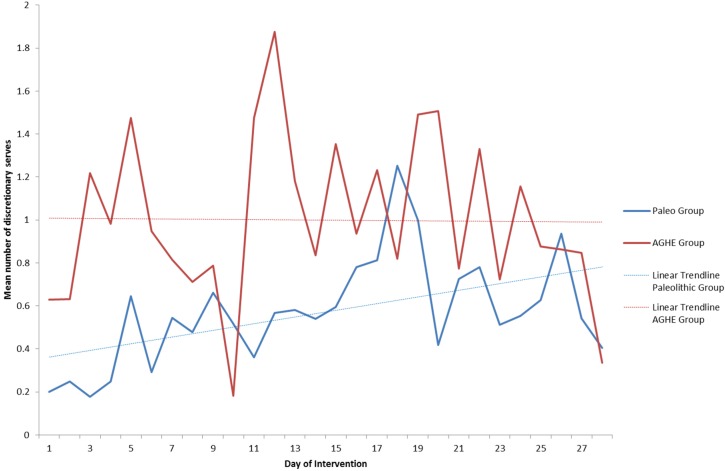
Compliance by day of intervention as measured by discretionary serve consumption.

The mean number of daily discretionary serves during the study was almost two fold higher in the AGHE compared to the Paleolithic group (Table 1). The mean consumption of extras varied from day to day between 0.2 and 1.8 with a trend towards an increase over the 4 weeks in the Paleolithic group, and a higher but more consistent consumption in the AGHE group (Figure 1). One subject in the Paleolithic group reported mean discretionary item consumption of 2.5 serves/day, while all other participants reported mean values below 2.0 serves/day.

### 3.2. Adverse Events

There were no significant differences in reported adverse events (Table 2) between groups except for diarrhoea, which was significantly higher in the Paleolithic group compared to AGHE group (*p* = 0.046). There were trends towards increased tiredness (*p* = 0.09), food cravings (*p* = 0.09) and increased trouble sleeping (*p* = 0.09) in the Paleolithic group compared to AGHE group.

### 3.3. Palatability and Feasibility Questionnaire

Of the Paleolithic respondents, 76% viewed the diet as healthy, compared with 93.8% in the AGHE group (NS) (Table 3) although a significantly greater proportion of the Paleolithic group reported that the diet did not fit with their belief of a healthy diet (*p* = 0.005). Both groups reported similar responses (*p* > 0.05) to: adapting to the dietary pattern, difficulty level after developing routines, levels of satisfaction after meals, overall taste, eating socially, overall health and wellbeing and shopping for appropriate foods. A significantly greater proportion of the Paleolithic group reported the cost of their food increased over the intervention period (*p* = 0.004).

## 4. Discussion

This study assessed the palatability and feasibility of the Paleolithic and AGHE diets, and compliance during a 4-week intervention in a group of 39 older women. The results showed high compliance to both dietary patterns, although we observed a trend towards an increased consumption of foods outside the Paleolithic dietary plan over the course of the intervention period. Outside the setting of a research intervention, the Paleolithic diet is likely to be followed by those with belief in the health benefits of the dietary pattern. However, the elimination of two food groups may be unsustainable over a longer period of time, with reports of cravings for restricted food identified as a barrier to following the diet after a period of time [8]. Urinary nitrogen analysis from a 2-year intervention using obese, post-menopausal women, who were assigned to either the Paleolithic (30% of energy from protein) or the Nordic Nutrition diet (15% of energy from protein), revealed no difference in protein intake, within or between groups at 6 and 24 months, indicative of poor compliance to target intakes [7]. We also reported a trend towards increased food cravings in the Paleolithic group, which could reflect difficulties in maintaining the dietary restrictions required. In contrast, the AGHE group reported a higher, but more consistent discretionary food consumption which aligned with the recommendations of 0–2.5 serves/day [3]. Our results of discretionary food intake are reflective of the guidelines of each of the dietary patterns, with recommended restriction of items containing added sugars, grains, dairy and legumes under a Paleolithic diet, but an allowance for some discretionary consumption under the AGHE. Both groups reported that eating out in social situations was difficult, suggesting that maintaining dietary restrictions may be problematic irrespective of the dietary regimen.

While both groups viewed the diets as healthy, a greater proportion of the Paleolithic group felt that the diet did not fit with the belief of a ‘healthy’ diet. This may reflect participant belief that the while the Paleolithic diet is high in ‘healthy foods’ such as fruits, vegetables, eggs, meats and nuts, the elimination of grains and dairy products makes the dietary pattern less healthy or unhealthy.

There is insufficient qualitative data on potential adverse effects of those following Paleolithic, or moderately low-carbohydrate style diets. Our finding of increased reported diarrhoea in the Paleolithic group was unexpected and may be due to changes in sub-fractions of fibre consumption, in turn impacting the gut microbiota [12]. Strict avoidance of grains and cereals and legumes means that the Paleolithic diet is intrinsically lower in carbohydrate than the AGHE, which includes these foods. A low carbohydrate intake in our study may have contributed to the observed trends towards increased tiredness, trouble sleeping and food cravings, although we note the study was not sufficiently powered in this area to detect statistically significant differences. Despite this, our findings are in line with other studies using low carbohydrate diets (<20–25 g/day), where participants have reported physical side effects such as weakness or headaches [13,14].

A significantly greater proportion of the Paleolithic group reported an increased cost of groceries, which was expected with the removal of low cost per kilogram grains and cereal products and replacement with more expensive items, fruits, vegetables, meat and nuts. Swedish women also reported increases in cost of groceries following a Paleolithic diet [8], which is in line with modelling estimations performed in the US by Metzgar, Rideout, Fontes-Villalba & Kuipers [10]. For the group following the AGHE diet, 94% of participants reported groceries were cheaper or cost the same, providing support for current public health interventions using the AGHE. Future research using the Paleolithic diet may need to consider cost when formulating study design. Further, as our study was conducted in a group of women, our findings cannot be extrapolated to the general population, and we recommend larger and longer duration interventions, including both men and women, for future research.

## 5. Conclusions

Compliance to both dietary regimens was high in the current study, however there is potential for increased side effects and cost on a Paleolithic diet, which may indicate the diet is impractical for use in clinical and public health settings. The AGHE intervention group rated the diet as healthy, had more consistent compliance and found the cost of groceries to be the same or more economical than their usual diet, providing support for use in future public health and clinical interventions.

## Figures and Tables

**Table 1:** Compliance assessment using number of serves of extras food per person.

	Paleolithic (*n* = 22) Mean ± SD	AGHE (*n* = 17) Mean ± SD	*p*-Value ^1^
Mean total extras over the 28-day intervention	15.9 ± 16	28.0 ± 17	0.03
Mean extras per day	0.569 ± 0.6	0.998 ± 0.6	0.03

^1^ Independent *t*-test.

**Table 2:** Proportion of participants who reported adverse events by dietary intervention group.

Adverse Event	Paleolithic (*n* = 22)	AGHE (*n* = 17)	Fisher’s Exact *p*-Value
Tiredness	15 (68.2%)	7 (41.2%)	0.09
Low Mood	2 (9.1%)	3 (17.6%)	0.38
Irritability	2 (9.1%)	0 (0%)	0.31
Irregular Bowel/Constipation	4 (18.2%)	2 (11.8%)	0.46
Diarrhoea	5 (22.7%)	0 (0%)	0.046 *
Hungry	8 (36.4%)	4 (23.5%)	0.39
Muscle cramps	3 (13.6%)	1 (6.2%)	0.41
Headache	8 (36.4%)	4 (23.5%)	0.39
Bloating	3 (13.6%)	3 (17.6%)	0.53
Thirsty	1 (4.5%)	0 (0%)	0.56
Trouble sleeping	4 (18.2%)	0 (0%)	0.09
Dizziness	4 (18.2%)	1 (6.2%)	0.26
Nausea	2 (9.1%)	0 (0%)	0.31
Food Cravings	4 (18.2%)	0 (0%)	0.09

* *p*-value < 0.05.

**Table 3:** Proportion responses collated from Palatability and Feasibility Questionnaire.

Question	Responses *n* (%)			*p*-Value ^†^
*How did you view the dietary pattern you were placed on?*		Healthy	Neutral or Unhealthy	0.21
	Paleo	16 (76.2%)	5 (23.8%)	
	AGHE	15 (93.8%)	1 (6.2%)	
*Did you find your dietary pattern fit with your belief of a healthy diet?*		Yes	Unsure or No	0.005 *
	Paleo	12 (57.1%)	9 (42.9%)	
	AGHE	16 (100%)	0 (0%)	
*Did you find this way of eating difficult to adapt to?*		Very easy or easy	Difficult or very difficult	0.46
	Paleo	14 (66.7%)	7 (33.3%)	
	AGHE	13 (81.2%)	3 (18.8%)	
*Did you feel like this way of eating was difficult once you had developed some routines with meals by the end of the study?*		Very easy, easy or okay	Difficult or very difficult	0.21
	Paleo	16 (76.2%)	5 (23.8%)	
	AGHE	15 (93.8%)	1 (6.2%)	
*How would you rate your level of satisfaction or fullness after most meals?*		Very full or full	Partly satisfied, still hungry, very hungry	1.00
	Paleo	17 (80.9%)	4 (19.1%)	
	AGHE	13 (81.2%)	3 (18.8%)	
*How would you rate the overall taste or palatability compared with your usual diet?*		Very tasty, tasty or okay	A little unpleasant, very unpleasant	0.12
	Paleo	17 (80.9%)	4 (19.1%)	
	AGHE	16 (100%)	0 (0%)	
*Did you find it easy to cope with situations such as eating out or other social gatherings?*		Very easy, easy or okay	Hard or very hard	0.52
	Paleo	13 (61.9%)	8 (38.1%)	
	AGHE	8 (50.0%)	8 (50.0%)	
*Do you feel like your desire to eat sugary foods has decreased after following this dietary pattern for a month?*		Completely, somewhat	About the same or not really	0.09
	Paleo	16 (76.2%)	5 (23.8%)	
	AGHE	7 (43.8%)	9 (56.2%)	
*How would you rate your general level of health and wellbeing after your change of diet?*		Feel much better, better	About the same, worse	0.73
	Paleo	14 (66.7%)	7 (33.3%)	
	AGHE	9 (56.2%)	7 (43.8%)	
*How easy did you find shopping for appropriate foods to eat from your daily checklist?*		Very easy, easy, okay	Difficult, very difficult	1.0
	Paleo	19 (90.5%)	2 (9.5%)	
	AGHE	15 (93.8%)	1 (6.2%)	
*Did you notice any changes to the cost of your grocery shopping over the past month?*		Much cheaper, cheaper, same	More expensive, much more expensive	0.004 *
	Paleo	10 (47.6%)	11 (52.4%)	
	AGHE	15 (93.8%)	1 (6.2%)	
*Do you feel like you would recommend this style of eating to your family or friends?*		Highly recommend, recommend	Maybe not, definitely not	0.46
	Paleo	14 (66.7%)	7 (33.3%)	
	AGHE	13 (81.2%)	3 (18.8%)	

^†^ Fishers exact test; * *p*-value < 0.05.

## Abbreviations

The following abbreviation is used in this manuscript:

AGHE	Australian Guide to Healthy Eating
